# Word-by-word entrainment of speech rhythm during joint story building

**DOI:** 10.3389/fpsyg.2015.00797

**Published:** 2015-06-12

**Authors:** Tommi Himberg, Lotta Hirvenkari, Anne Mandel, Riitta Hari

**Affiliations:** Brain Research Unit, Department of Neuroscience and Biomedical Engineering, School of Science, Aalto UniversityEspoo, Finland

**Keywords:** turn-taking, entrainment, word rhythm, mutual adaptation, speech, social interaction

## Abstract

Movements and behavior synchronize during social interaction at many levels, often unintentionally. During smooth conversation, for example, participants adapt to each others' speech rates. Here we aimed to find out to which extent speakers adapt their turn-taking rhythms during a story-building game. Nine sex-matched dyads of adults (12 males, 6 females) created two 5-min stories by contributing to them alternatingly one word at a time. The participants were located in different rooms, with audio connection during one story and audiovisual during the other. They were free to select the topic of the story. Although the participants received no instructions regarding the timing of the story building, their word rhythms were highly entrained (øverlineR = 0.70, *p* < 0.001) even though the rhythms as such were unstable (øverlineR = 0.14 for pooled data). Such high entrainment in the absence of steady word rhythm occurred in every individual story, independently of whether the subjects were connected via audio-only or audiovisual link. The observed entrainment was of similar strength as typical entrainment in finger-tapping tasks where participants are specifically instructed to synchronize their behavior. Thus, speech seems to spontaneously induce strong entrainment between the conversation partners, likely reflecting automatic alignment of their semantic and syntactic processes.

## Introduction

During human social interaction, body movements and behavior synchronize at many levels. This interpersonal coordination can be intentional or unintentional, and it can take many shapes. In conversation, participants' utterance length, vocabulary, and information density, as well as body posture and the use of non-verbal gestures often adapt or match (Condon and Ogston, 1967; Kendon, 1970; Giles et al., 1991; Chartrand and Bargh, 1999; Gonzales et al., 2010). Similarly, continuous rhythmic behaviors can entrain, or converge in phase and period (Pikovsky et al., 2001; Clayton et al., 2004). Such an entrainment to a common rhythm can be seen in music and dance, finger tapping, rocking in chairs or gait when walking side by side (Boker et al., 2005; Repp, 2005; Richardson et al., 2007; Nessler and Gilliland, 2009; Himberg and Thompson, 2011). Entrainment has positive affective consequences (Hove and Risen, 2009; Wiltermuth and Heath, 2009), and while foregrounded in music and dance, timing and entrainment also play important roles in verbal and non-verbal communication (Bavelas et al., 1986; Shockley et al., 2003; Cummins, 2009).

During smooth conversation, turn-taking is accurately regulated between the participants, who thereby can avoid overlap of speech and optimize silence between the turns. To time their own contributions correctly, the participants need to be able to predict the end of their partner's turn. Traditionally, turn-taking is said to be governed by a set of linguistic rules (Sacks et al., 1974), while more contemporary theories have suggested turn-taking to be driven by entrainment of oscillatory processes (Wilson and Wilson, 2005), and to operate at the level of prosody and timing, rather than linguistic units (Cowley, 1998). Turn-taking is often seen as fundamental in human cognition, even as a species-specific, evolutionary adaptation (Sidnell, 2001). The basic mechanisms of turn-taking are thought to be universal, although different languages somewhat vary in the optimal duration of gaps between turns (Stivers et al., 2009).

Interpersonal entrainment is a result of continuous mutual adaptation, as has been demonstrated in simple hand-tapping tasks performed by two persons (Konvalinka et al., 2010) as well as in dance (Himberg and Thompson, 2011). Such mutual adaptation emerges in live dyadic interaction and can be observed already in infants (Malloch and Trevarthen, 2009). For example, when participants read texts together, their verbal outputs are better synchronized when they are in live interaction than when they co-read with recorded speech (Cummins, 2009). Moreover, partners synchronize their finger-tapping better with other humans than with non-responsive computer partners (Himberg, 2014).

Interpersonal coordination in dyads and groups can either occur by matching behaviors, such as gestures, posture, or vocabulary, or as continuous synchronization (Bernieri and Rosenthal, 1991; Dale et al., 2013). Both types of coordination occur in natural conversations, but from an experimental perspective, both have complications. Behavior matching, although commonly observed in many aspects of conversations (e.g. as imitation of the other person's actions, called “chameleon effect” by Chartrand and Bargh, 1999), occurs intermittently, as the interlocutors do not mirror each other's contributions, but rather interact in a complementary fashion (Abney et al., 2014). Also, the time lags of matching are unpredictable, and can be as long as minutes (Louwerse et al., 2012). Continuous synchronization also occurs during natural conversations, for example, the body sways of the interlocutors synchronize. However, these movements are so small that measuring them requires special sensors, and even then the signal is noisy and the data analysis is complicated (Shockley et al., 2003). To overcome these complications, we used a word game where turns change predictably and often enough, and thus we could measure interpersonal coordination from the speech signals.

Our aim was to experiment on interpersonal coordination using a linguistic task, to contrast with the cognitively less challenging finger-tapping tasks that are the traditional approaches to studying intentional synchrony (Repp, 2005). We aimed at a task that would feel natural and be easy to explain to the subjects and would allow us to measure interpersonal synchronization directly from the speech signals, rather than relying on changes in secondary, oscillatory movements, such as swinging a pendulum or rocking in a chair (Richardson et al., 2005, 2007). Unlike Reich et al. (2014) who looked at pitch synchrony between therapists and clients, we were interested in word timing. We thus asked pairs of participants to create stories word by word, each contributing one word at a time. Since turn-taking occurred after every word, we were able to study word timing in a relatively controlled situation. As Finnish is a highly inflected language, each turn consisted of a meaningful word, rather than a preposition, article etc. that do not exist in Finnish (see Supplementary Information 1). Our participants were seated in separate rooms and connected via either an audiovisual link (“video call”) or audio-only link (“telephone call”), allowing us to analyze the relative contributions of auditory and visual cues to speech-rhythm entrainment. The terminology and criteria regarding synchronization and entrainment vary largely in the literature (for a review, see Himberg, 2014, pp. 21–35), but in the present study, by word-rhythm entrainment, we refer to phase-locking of the temporal sequences of word onset times of the two participants.

## Methods

### Participants, apparatus, materials

We studied 18 healthy adults (12 males, 6 females; aged 21–43 years, mean ± SD 27.1 ± 0.6 years), all native Finnish speakers, forming 9 sex-matched pairs. After the course of the study had been explained to the subjects, they gave their written informed consent. The study had prior approval by the Ethics Committee of the Hospital District of Helsinki and Uusimaa.

The data were collected during a two-person magneto-encephalography (MEG) experiment, using a MEG2MEG setup (Baess et al., 2012) but only the behavioral results will be reported here. Participants were seated in separate rooms and, depending on the task condition, they had either an audio-only connection (microphones and headphones), or an audiovisual connection where they could also see a video feed of the other participant in natural size on a projection screen positioned 1 m in front of them.

In our custom-made internet-based communication system, the one-way latency is 50 ± 2 ms for audio signal and 130 ± 12 ms for video (Zhdanov et al., in press). In a pilot dyad, the participants reported they did not notice any lags in either audio or video transmission, and they rated the feeling of presence of their partner at 9 on a 10-point scale. Our participants also reported not to have detected the 80-ms asynchrony between the audio and video inputs during normal conversation that was also included in the setup. This feeling of real-life-like presence of the other person is understandable because the audio and video latencies of our system were well under the limits for smooth conversation (100 ms for audio, 500 ms for video; Jansen and Bulterman, 2013), and even under the limits for more delay-sensitive tasks (60 and 140 ms; Kurita et al., 1994). The asynchrony between the audio and video inputs was within the 130-ms integration window within which auditory and visual speech inputs are considered synchronous, when the auditory input precedes the visual one (Dixon and Spitz, 1980; Vroomen and Stekelenburg, 2011). We therefore considered the transmission latencies of our setup to be negligible for our task, where inter-word intervals were over 2 s.

### Procedure

Participants were instructed to construct a story, contributing one word at a time in alternating turns. They were free to select the topic of the story, and no instructions were given regarding the rhythm or the timing of the words. The experimenter indicated which participant was supposed to start. The stories were about 5 min in duration. Each dyad constructed two stories, one in which they had only an audio connection, and another where they also could see each other on screen. The order of conditions was counterbalanced across dyads. Because of time constraints, two of the nine dyads completed the task in only one of the two conditions, leaving 16 stories to be analyzed.

### Analysis

We aimed to quantify the rhythm of speech and the interdependence of word timing both for each single individual and between the participants of a dyad. A total of seven instances of coughing, laughing and interruptions due to not hearing the word were removed from the data.

In speech, the stream of stressed syllables generates the word rhythm (Vos et al., 1995; Scott, 1998). In Finnish, word stress occurs on the first syllable of the word (Iivonen, 1998), and therefore we opted to use word onsets as the basis of our word-rhythm analysis. Word onset and offset times were defined in Matlab 7 (MathWorks) from the 48-kHz audio files as the moments where the sound envelope exceeded the level of background noise during silence. After the detection of the onset and offset times, each sound was labeled manually as a word or a non-word and then transcribed. If the actual word was preceded by an interjection (participant saying e.g. “umm… fishing”), the beginning of the interjection was selected as the onset time, as in many cases the interjection and the word were inseparably merged.

**Figure 1:**
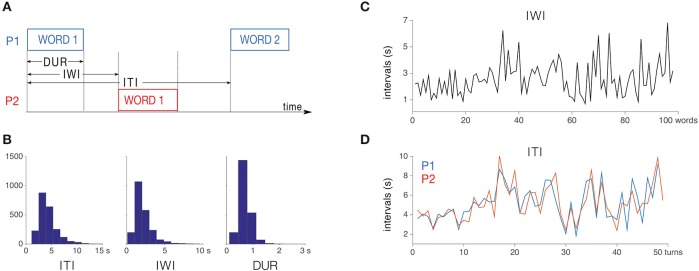
**(A)** variables extracted from the word onset-offset times; **(B)** histograms of inter-turn intervals, inter-word intervals and word durations; **(C)** IWI, the joint series of word timings, example data from one story; **(D)** ITI, the individual series of word timings, example data from the same story as in **(C)**. Blue line refers to Participant 1 and red line to Participant 2.

Four different time series were extracted from the word-onset and -offset data (Figure 1A): inter-turn intervals (ITIs; times between consecutive word onsets for one speaker), inter-word intervals (IWIs; times between successive word onsets in the joint stream), word durations (DURs), and gap durations (GAPs).

The IWI and ITI time series were converted to phase values, and the concentrations of the resulting circular distributions were used as stability and entrainment measures (see Supplementary Information 2). The stability measure represented the “steadiness” of the consecutive IWIs or ITIs, quantifying how similar each time interval was in relation to the previous one: equally long intervals yielded a phase value of zero, while deviations yielded non-zero values ranging from 1 to 359°.

The entrainment measure, on the other hand, reflected the consistency of the interrelationship between the ITIs of the two participants. It was calculated by measuring where, within one participant's ITIs, the other participant's word onsets occurred. If Participant 1 uttered a word at exactly half way the ITI of Participant 2, the phase value was 180°, with deviations from this anti-phase state ranging theoretically from 0 to 359°. In practice, however, the possible range of relative angles was somewhat narrower (we observed it to range from 14 to 326°), because the participants needed to avoid overlaps (zero relative phase would mean that both participants would start their words simultaneously).

For both stability and entrainment, circular distribution measure R (Fisher, 1993, p. 32) and mean angle θ were calculated for each trial, as well as for all the data of the experiment. R ranges from 0 (no stability or no entrainment) to 1 (perfect stability or perfect entrainment), and it has previously been used in quantifying individual timing stability and especially synchronicity and entrainment in dyadic and group timing (Himberg, 2006, 2014; Rankin et al., 2009; Lucas et al., 2011).

To statistically evaluate whether the word rhythms in trials were stable and/or entrained, we conducted *V*-tests and Kuiper two-sample tests to see if the observed distributions statistically differed from uniform distributions (Fisher, 1993; Jammalamadaka and Sengupta, 2001). For the entrainment measure, we compared the observed distribution with a uniform distribution from 14 to 326°, corresponding to the range of phase angles that was observed in the study.

## Results

### General

In the 16 stories by 9 different dyads, a total of 2261 words were uttered, on average 141.3 words per story, or 70.7 (range 43–110) words per participant per story. Figure 1B shows the histograms for word durations, IWIs, and ITIs. The word durations were on average (mean ± SD) 0.69 ± 0.23 s, IWIs were 2.14 ± 1.15 s, and ITIs were 4.29 ± 1.80 s.

Participants produced the words in a normal tempo with a mean rate of 3.3 syllables/s, which is comparable to that of normal spoken Finnish (Toivola et al., 2009). As expected, due to the nature of the task, the gaps between words (on average 1.45 s) were longer than in normal, continuous speech (0.5 s, Toivola et al., 2009). The sentences that the participants constructed together were syntactically coherent. Across all pairs, sentences contained on average 9.3 ± 1.7 words (range 2–24), and a story contained on average 14.9 ± 6.8 sentences.

### Stability and entrainment

Figures 1C,D show the ITI and IWI data from an individual story. Both the IWIs and ITIs varied a lot from one word to the next, often by several seconds, making word timing unstable. However, the inter-turn intervals of the two participants (1D) were highly correlated (for this example *r* = 0.72, *p* < 0.001) with each other, indicating high entrainment between the participants.

**Figure 2:**
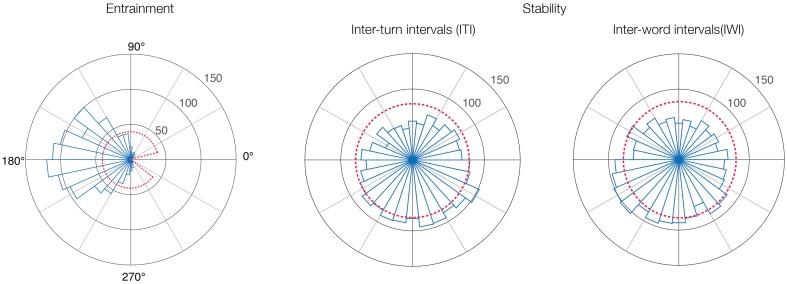
**High entrainment in the absence of stability**. Circular histograms of relative phase (entrainment) and stability distributions in the whole experiment. Red dashed lines represent uniform distributions of data and the range of observed data.

The circular histograms in Figure 2 confirm this pattern for the whole experiment, demonstrating that word rhythms were highly entrained even though the individual and joint timings were unstable. The distribution of the relative phase angles (Figure 2) calculated from the ITIs has a clear preferred direction toward 180°, indicating anti-phase entrainment. The entrainment measure for the pooled data was R = 0.70, and R = 0.74 ± 0.05 for the 16 individual stories. Instead of varying evenly within its observed range (14–326°), the distribution shows a heavy weighting to anti-phase angles, with 95% of the values concentrated between 78 and 270°. This phase attraction toward the anti-phase was also demonstrated in statistical tests, where, in all stories, the observed distributions deviated statistically significantly from uniform distributions (*p* < 0.01; Kuiper test).

In contrast, the distributions for both the individual word timings (ITI, Figure 2) and in the joint time series of word onsets (IWI) were uniformly spread around the circle without any clear preference. The stability measures were very low, R = 0.14 for ITI, and R = 0.13 for IWI.

Looking at the 16 joint time series and the 32 individual time-series (16 stories ^*^ 2 participants) separately, the stability measure R was 0.15 ± 0.064 for the joint timings (IWIs) and 0.18 ± 0.071 for the

individual timings (ITIs). *V*-tests confirmed that with the exception of three cases, all individual ITI time-series were unstable, as the distributions did not differ from uniform distribution toward the expected mean direction of zero at *p* < 0.05.

The stability and entrainment scores did not differ between the audio-only and audiovisual conditions (*p* = 0.39 for IWI, *p* = 0.15 for ITI, and *p* = 0.15 for entrainment; paired two-tailed *t*-tests).

## Discussion

We found that when two participants were creating stories together, in turns, one word at a time, their word rhythms were strongly entrained. Such a high level of entrainment was unexpected, as the word rhythms themselves were very unstable, and the participants were not given any instructions related to word rhythm, tempo, or timings of their words. Previously, entrainment of comparable strength has been observed in finger-tapping tasks, where the entraining beats occur at equal intervals and the participants are specifically asked to aim for accurate anti-phase timing. The unexpected independence of high levels of entrainment from a stable word-to-word rhythm is in line with the oscillation-based theory of turn-taking (Wilson and Wilson, 2005), which assumes that conversation participants are entrained to a common rhythm that is established by shared syllable timing (Street, 1984). This shared rhythm governs the participants' “readiness” to take turns, and it helps them to optimize turn-taking so that it does not comprise overlaps and long silences.

Interpersonal coordination and adaptation occur in a wide range of tasks, such as pronouncing letters of the alphabet (Kawasaki et al., 2013) or in anti-phase finger tapping (Nowicki et al., 2013). These rather simple tasks mainly recruit automatic entrainment processes, whereas our task of joint story building required advanced cognitive operations to guarantee that the story evolved in a meaningful and smooth manner.

As an automatic and subconscious process, entrainment is assumed to subserve communicative interaction (Gallese, 2001, 2005; Himberg, 2014), and in our task, participants needed to be aligned at the semantic and syntactic levels, as well as the speech-process level (Clark, 1996; Garrod and Pickering, 2004). The high entrainment that we observed could be what allowed the participants to reach this multi-level, multimodal coordination (Dale et al., 2013).

In our study, stability and entrainment were statistically similar in “telephone-like” trials (with only auditory connection between the participants) and “video-call-like” trials (with auditory and visual connection between the participants). This result partly agrees with the results of a previous corpus study of face-to-face as well as telephone dialogs, where pause durations between participants were highly correlated in both types of conversations, suggesting entrainment to a common rhythm even in telephone-mediated conversations (Ten Bosch et al., 2004). However, in the corpus study, the pauses were longer and more variable in the face-to-face conversations. We did not observe such differences, possibly because due to the simultaneous MEG recording, our participants were asked to sit still, which limited the amount and utility of gestural communication between them. All task-critical information was delivered through the auditory channel.

Although our participants conducted the story-building task in a laboratory setting that restricted their body movements, highly entrained speech rhythms emerged spontaneously in their interaction. This mutual adaptation of speech rhythms implies speech as a strong inducer of entrainment, even when the participants just hear each other.

### Conflict of interest statement

The authors declare that the research was conducted in the absence of any commercial or financial relationships that could be construed as a potential conflict of interest.

## References

[B1] Abney D. H. Paxton A. Dale R. Kello C. T. (2014). Complexity matching in dyadic conversation. J. Exp. Psychol. Gen. 143, 2304–2318. 10.1037/xge0000021 25285431

[B1a] Baess P. Zhdanov A. Mandel A. Parkkonen L. Hirvenkari L. Mäkelä J. P.. (2012). MEG dual scanning: a procedure to study real-time auditory interaction between two persons. Front. Hum. Neurosci. 6, 83. 10.3389/fnhum.2012.00083 22514530 PMC3322488

[B2] Bavelas J. Black A. Lemery C. Mullett J. (1986). I show how you feel: motor mimicry as a communicative act. J. Pers. Soc. Psychol. 50, 322–329. 10.1037/0022-3514.50.2.322

[B3] Bernieri F. J. Rosenthal R. (1991). Interpersonal coordination: behavior matching and interactional synchrony, in Fundamentals of Nonverbal Behavior, eds Feldman R. S. Rimé B. (Cambridge: Cambridge University Press), 401–432.

[B4] Boker S. Covey E. Tiberio S. Deboeck P. (2005). Synchronization in dancing is not winner-takes-all: Ambiguity persists in spatiotemporal symmetry between dancers, in Proceedings of the North American Association for Computational, Social, and Organizational Science (Notre Dame, IN).

[B5] Chartrand T. Bargh J. (1999). The chameleon effect: the perception-behavior link and social interactions. J. Pers. Soc. Psychol. 76, 893–910. 10.1037/0022-3514.76.6.893 10402679

[B6] Clark H. H. (1996). Using Language. Cambridge: Cambridge University Press.

[B7] Clayton M. Sager R. Will U. (2004). In time with the music: The concept of entrainment and its significance for ethnomusicology. ESEM Counterpoint 1, 1–45. Available online at: http://oro.open.ac.uk/id/eprint/2661

[B8] Condon W. Ogston W. (1967). A segmentation of behavior. J. Psychiatr. Res. 5, 221–235. 10.1016/0022-3956(67)90004-0

[B9] Cowley S. J. (1998). Of timing, turn-taking, and conversations. J. Psycholinguist. Res. 27, 541–571. 10.1023/A:1024948912805

[B10] Cummins F. (2009). Rhythm as entrainment: the case of synchronous speech. J. Phon. 37, 16–28. 10.1016/j.wocn.2008.08.003

[B11] Dale R. Fusaroli R. Duran N. Richardson D. C. (2013). The self-organization of human interaction. Psychol. Learn. Motiv. 59, 43–95. 10.1016/b978-0-12-407187-2.00002-2

[B12] Dixon N. F. Spitz L. (1980). The detection of auditory visual desynchrony. Perception 9, 719–721. 10.1068/p090719 7220244

[B13] Fisher N. (1993). Statistical Analysis of Circular Data. Cambridge: Cambridge University Press.

[B14] Gallese V. (2001). The shared manifold hypothesis. From mirror neurons to empathy. J. Conscious. Stud. 8, 33–50. 14504450

[B15] Gallese V. (2005). Embodied simulation: from neurons to phenomenal experience. Phenomenol. Cogn. Sci. 4, 23–48. 10.1007/s11097-005-4737-z

[B16] Garrod S. Pickering M. (2004). Why is conversation so easy? Trends Cogn. Sci. 8, 8–11. 10.1016/j.tics.2003.10.016 14697397

[B18] Giles H. Coupland N. Coupland J. (1991). Accommodation theory: communication, context, and consequence, in Contexts of Accommodation: Developments in Applied Sociolinguistics, eds Giles H. Coupland N. Coupland J. (Cambridge: Cambridge University Press), 1–68.

[B19] Gonzales A. L. Hancock J. T. Pennebaker J. W. (2010). Language style matching as a predictor of social dynamics in small groups. Communic. Res. 37, 3–19. 10.1177/0093650209351468

[B20] Himberg T. (2006). Co-operative tapping and collective time-keeping - differences of timing accuracy in duet performance with human or computer partner, in Proceedings of the ICMPC 9, eds Baroni M. Addessi A. R. Caterina R. Costa M. (Bologna), 377.

[B21] Himberg T. (2014). Interaction in Musical Time. Doctoral dissertation, Faculty of Music, University of Cambridge, Cambridge.

[B22] Himberg T. Thompson M. R. (2011). Learning and synchronising dance movements in South African songs – cross-cultural motion-capture study. Dance Res. 29, 305–328. 10.3366/drs.2011.0022

[B23] Hove M. J. Risen J. L. (2009). It's all in the timing: interpersonal synchrony increases affiliation. Soc. Cogn. 27, 949–960. 10.1521/soco.2009.27.6.949

[B24] Iivonen A. (1998). Intonation in Finnish, in Intonation Systems: A Survey of Twenty Languages, eds Hirst D. Di Cristo A. (Cambridge: Cambridge University Press), 311–327.

[B25] Jammalamadaka S. R. Sengupta A. (2001). Topics in Circular Statistics, Vol. 5 Singapore: World Scientific Publishing.

[B26] Jansen J. Bulterman D. C. (2013). User-centric video delay measurements, in Proceedings of the 23rd ACM Workshop on Network and Operating Systems Support for Digital Audio and Video (Oslo: ACM), 37–42.

[B27] Kawasaki M. Yamada Y. Ushiku Y. Miyauchi E. Yamaguchi Y. (2013). Inter-brain synchronization during coordination of speech rhythm in human-to-human social interaction. Sci. Rep. 3:1692. 10.1038/srep01692 23603749 PMC3631767

[B28] Kendon A. (1970). Movement coordination in social interaction: some examples described. Acta Psychol. 32, 101–125. 10.1016/0001-6918(70)90094-6 5444439

[B29] Konvalinka I. Vuust P. Roepstorff A. Frith C. (2010). Follow you, follow me: continuous mutual prediction and adaptation in joint tapping. Q. J. Exp. Psychol. 63, 2220–2230. 10.1080/17470218.2010.497843 20694920

[B30] Kurita T. Lai S. Kitawaki N. (1994). Effects of transmission delay in audiovisual communication. Electron. Commun. Jpn. 77, 63–74. 10.1002/ecja.4410770306

[B31] Louwerse M. M. Dale R. Bard E. G. Jeuniaux P. (2012). Behavior matching in multimodal communication is synchronized. Cogn. Sci. 36, 1404–1426. 10.1111/j.1551-6709.2012.01269.x 22984793

[B32] Lucas G. Clayton M. Leante L. (2011). Inter-group entrainment in Afro-Brazilian congado ritual. Empir. Musicol. Rev. 6, 75–102. Available online at: http://hdl.handle.net/1811/51203

[B33] Malloch S. Trevarthen C. (2009). Musicality: communicating the vitality and interests of life, in Communicative Musicality - Exploring the Basis of Human Companionship, eds Malloch S. Trevarthen C. (Oxford: Oxford University Press), 1–11.

[B34] Nessler J. A. Gilliland S. J. (2009). Interpersonal synchronization during side by side treadmill walking is influenced by leg length differential and altered sensory feedback. Hum. Mov. Sci. 28, 772–785. 10.1016/j.humov.2009.04.007 19796834

[B35] Nowicki L. Prinz W. Grosjean M. Repp B. H. Keller P. E. (2013). Mutual adaptive timing in interpersonal action coordination. Psychomusicol. Music Mind Brain 23, 6–20. 10.1037/a0032039

[B36] Pikovsky A. Rosenblum M. Kurths J. (2001). Synchronization - a Universal Concept in Non-linear Sciences (No. 12). Cambridge: Cambridge University Press.

[B37] Rankin S. K. Large E. W. Fink P. W. (2009). Fractal tempo fluctuation and pulse prediction. Music Percept. 26, 401–413. 10.1525/mp.2009.26.5.401 25190901 PMC4151502

[B38] Reich C. M. Berman J. S. Dale R. Levitt H. M. (2014). Vocal synchrony in psychotherapy. J. Soc. Clin. Psychol. 33, 481–494. 10.1521/jscp.2014.33.5.481

[B39] Repp B. H. (2005). Sensorimotor synchronization: a review of the tapping literature. Psychon. Bull. Rev. 12, 969–992. 10.3758/BF03206433 16615317

[B40] Richardson M. J. Marsh K. L. Schmidt R. C. (2005). Effects of visual and verbal interaction on unintentional interpersonal coordination. J. Exp. Psychol. Hum. Percept. Perform. 31, 62–79. 10.1037/0096-1523.31.1.62 15709863

[B41] Richardson M. Marsh K. Isenhower R. Goodman J. Schmidt R. (2007). Rocking together: dynamics of intentional and unintentional interpersonal coordination. Hum. Mov. Sci. 26, 867–891. 10.1016/j.humov.2007.07.002 17765345

[B42] Sacks H. Schegloff E. A. Jefferson G. (1974). A simplest systematics for the organization of turn-taking for conversation. Language 696–735. 10.1353/lan.1974.0010

[B43] Scott S. K. (1998). The point of p-centres. Psychol. Res. 61, 4–11. 10.1007/PL00008162

[B44] Shockley K. Santana M.-V. Fowler C. A. (2003). Mutual interpersonal postural constraints are involved in cooperative conversation. J. Exp. Psychol. Hum. Percept. Perform. 29, 326–332. 10.1037/0096-1523.29.2.326 12760618

[B44a] Sidnell J. (2001). Conversational turn-taking in a Caribbean English Creole. J. Pragmatics 33, 1263–1290.

[B45] Stivers T. Enfield N. Brown P. Englert C. Hayashi M. Heinemann T.. (2009). Universals and cultural variation in turn-taking in conversation. Proc. Natl. Acad. Sci.U.S.A. 106, 10587–10592. 10.1073/pnas.0903616106 19553212 PMC2705608

[B46] Street R. L. (1984). Speech convergence and speech evaluation in fact-finding interviews. Hum. Commun. Res. 11, 139–169. 10.1111/j.1468-2958.1984.tb00043.x

[B47] Ten Bosch L. Oostdijk N. De Ruiter J. P. (2004). Durational aspects of turn-taking in spontaneous face-to-face and telephone dialogues, in Proceedings of the 7th International Conference Text, Speech and Dialogue, eds Sojka P. Kopecek I. Pala Brno K. (Berlin: Springer).

[B48] Toivola M. Lennes M. Aho E. (2009). Speech rate and pauses in non-native Finnish, in Proceedings of the 10th Annual Conference of the International Speech Communication Association. (Brighton).

[B49] Vos P. G. Mates J. van Kruysbergen N. W. (1995). The perceptual centre of a stimulus as the cue for synchronization to a metronome: evidence from asynchronies. Q. J. Exp. Psychol. 48, 1024–1040. 10.1080/14640749508401427 8559964

[B50] Vroomen J. Stekelenburg J. J. (2011). Perception of intersensory synchrony in audiovisual speech: not that special. Cognition 118, 75–83. 10.1016/j.cognition.2010.10.002 21035795

[B51] Wilson M. Wilson T. (2005). An oscillator model of the timing of turn-taking. Psychon. Bull. Rev. 12, 957–968. 10.3758/BF03206432 16615316

[B52] Wiltermuth S. Heath C. (2009). Synchrony and cooperation. Psychol. Sci. 20, 1–5. 10.1111/j.1467-9280.2008.02253.x 19152536

[B53] Zhdanov A. Nurminen J. Baess P. Hirvenkari L. Jousmäki V. Mäkelä J. P. (in press). An internet-based real-time audiovisual link for dual meg recordings. PLoS ONE. 10.1371/journal.pone.0128485 PMC4476621 26098628

