# Supplementary material for: Word-by-word entrainment of speech rhythm during joint story building
Source: Front Psychol. 2015 Jun 12;6:797. doi: 10.3389/fpsyg.2015.00797 (PMC4464109; doi:10.3389/fpsyg.2015.00797)
Supplement: Supplementary file 1 [file Presentation1.PDF]

frontiers

Supplementary Material

Word-by-word entrainment of speech rhythm during joint story building

Tommi Himberg[1]*, Lotta Hirvenkari[1], Anne Mandel[1], Riitta Hari[1]

[1]Department of Neuroscience and Biomedical Engineering, School of Science, Aalto University, Espoo, Finland

*** Correspondence:** Tommi Himberg, Department of Neuroscience and Biomedical Engineering, School of Science, Aalto University, Rakentajanaukio 2C, FI-02150, Espoo, Finland.
tommi.himberg@aalto.fi

1. Word game and the Finnish language

Finnish is a *highly inflected, agglutinative language*, where case, number, person and tense are expressed by adding relevant morphemes to the word stem. Thus, there are no separate prepositions or articles like in English, which makes Finnish naturally suitable for this type of a word game, where participants build stories taking turns, one word at a time. To take an example from our data, one dyad produced the following sentence (blue and red colours indicate the contributions of participants 1 and 2, respectively): *"Aamulla nainen heräsi sängystään ja huomasi, että vieressä ei ollutkaan ketään."* This translates into English as: *"In the morning, a woman woke up in her bed and noticed that next to her, there was not anybody."* As the colouring indicates, in English up to three words are needed when in Finnish the same meaning is synthesised in a single word. Thus in Finnish, each word will be a meaningful "chunk" of the story, and thus the instruction to contribute one word per turn is easily understood and unambiguous. In contrast, were the game to be played in English, the participants would have to agree whether e.g. "in" or "the" would count as a word, or should each participant contribute a more complete phrase (such as "in the morning") at a time, instead of just an individual word. Using phrases as turn units would likely influence the interpersonal rhythms between participants.

2. Calculating the stability and entrainment measures

To calculate the stability and entrainment measures, the onset time series were first converted into phase values. Then, concentration measures of the circular distributions of these phase angles were calculated and used as stability and entrainment measures (Fisher, 1993, 31–32). In both cases, the phase values were computed using the stroboscopic observation method (Pikovsky et al., 2001, 163), where the linearly growing phase of the baseline is observed at the times of the onset of the signal. The phase value computations for the stability and entrainment measures are similar, only the choice of baseline differs.

When calculating he stability measure, the previous inter-onset intervals of either the individual ITI time series or the joint IWI time series served as the baseline (see Supplementary Figure 1A). The interval observed between word onsets 1 and 2 was projected forward, and then the phase of this projection was observed at the time of onset of word 3. When calculating the entrainment measure, the ITIs of one participant served as the baseline, the phase of which was observed at the times of the word onsets of the other participants (Supplementary Figure 1B). In both cases, the phase of the baseline was set to grow linearly within each cycle, from 0 to 2π, then resetting to zero.

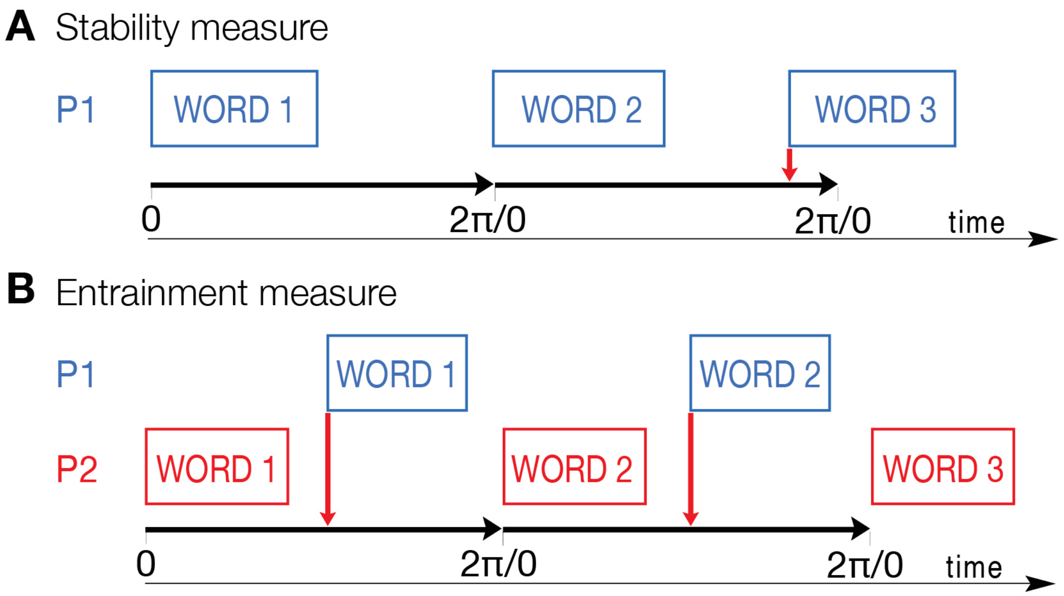

Supplementary Figure 1. Converting onset times to phase values when computing the stability and entrainment measures. (A) When computing the stability measure, each inter-onset interval was compared with the previous one. (B) The onsets of one of the participants (in this case, P1) were converted to phase values by observing where along the linearly growing phase of the other participant (here P2) they occurred.
